# Pheromonal variation and mating between two mitotypes of fall armyworm (*Spodoptera frugiperda*) in Africa

**DOI:** 10.1038/s41598-024-53053-9

**Published:** 2024-02-15

**Authors:** Birhanu Sisay, Amanuel Tamiru, Sevgan Subramanian, Christopher W. Weldon, Fathiya Khamis, Kristina Karlsson Green, Peter Anderson, Baldwyn Torto

**Affiliations:** 1 International Centre of Insect Physiology and Ecology (Icipe), P.O. Box 30772-00100, Nairobi, Kenya; 2 Department of Zoology and Entomology, Forestry and Agriculture Biotechnology Institute, University of Pretoria, Private Bag X20, Hatfield, 0028 South Africa; 3 Ethiopian Institute of Agricultural Research, Melkassa Agricultural Research Centre, P.O. Box 436, Adama, Ethiopia; 4 Department of Plant Protection Biology, Swedish University of Agricultural Sciences, Alnarp, Box 190, 23422 Lomma, Sweden; 5 Department of Zoology and Entomology, University of Pretoria, Private Bag X20, Hatfield, 0028 South Africa

**Keywords:** Entomology, Invasive species, Agroecology

## Abstract

In the Americas, the fall armyworm (*Spodoptera frugiperda*) exists in two genetically distinct strains, the corn (C) and rice (R) strains. Despite their names, these strains are not associated with host plant preferences but have been shown to vary in pheromone composition and male responses. Recently, *S. frugiperda* was detected in Africa as an invasive species, but knowledge about variation in strain types, pheromone composition and inter-strain mating of populations of the pest in the continent has not been fully examined. Therefore, this study aimed to investigate variations, if any in the pheromone composition of female moths, male moth responses, and mating between C and R mitotypes of *S. frugiperda* populations in Kenya, as well as their geographic distribution. Strains (mitotypes) of *S. frugiperda* were identified using mitochondrial DNA (mtDNA) markers, and their pheromonal composition determined by coupled gas chromatography–mass spectrometric (GC–MS) analysis. Male moth responses to these compounds were evaluated using GC-electroantennographic detection (EAD), electroantennogram (EAG), and wind tunnel assays. Oviposition assays were used to determine whether R and C mitotype moths could mate and produce eggs. The results showed that both the R and C mitotypes were present, and there were no statistically significant differences in their distribution across all sampled locations. Five pheromone compounds including (*Z*)-7-dodecenyl acetate (*Z*7-12:OAc), (*Z*)-7-tetradecenyl acetate (*Z*7-14:OAc), (*Z*)-9-tetradecenyl acetate (*Z*9-14:OAc), (*Z*)-11-tetradecenyl acetate (*Z*11-14:OAc) and (*Z*)-11-hexadecenyl acetate (*Z*11-16:OAc), were detected in the pheromone glands of female moths of both mitotypes, with *Z*9-14:OAc being the most abundant. The relative percentage composition of *Z*9-14:OAc was similar in both mitotypes. However, the R mitotype had a 2.7 times higher relative percentage composition of *Z*7-12:OAc compared to the C mitotype moth, while the C mitotype moth had a 2.4 times higher relative percentage composition of *Z*11-16:OAc than the R mitotype moth. Male moths of both mitotypes exhibited similar responses to the pheromone compounds, showing the strongest responses to *Z*9-14:OAc and *Z*7-12:OAc in electrophysiological and behavioural assays. There was mating between R and C mitotypes with egg production comparable to mating within the same mitotype. Our results revealed that differences between the two *S. frugiperda* mitotypes are characterized by female moth pheromone composition rather than male moth responses to the pheromones, and that this does not prevent hybridisation between the mitotypes, which may have implications for their management.

## Introduction

Insects use chemical signals, known as semiochemicals, to communicate with members of their own species (intraspecific) as well as other species (interspecific)^1,2^. Pheromones are common intraspecific semiochemcials used by insects, including many lepidopteran species, where moths rely on sex pheromones produced by female moths to find mates for reproduction^3–5^. Male moths exhibit dramatic behavioural and physiological responses to these pheromones^6,7^. However, the sexual communication system of several lepidopteran species can vary widely across different geographic regions^8–15^, which may have consequences for gene flow between populations and eventually speciation.

Previous studies have shown that the *S. frugiperda*, exhibits geographic variation in sexual communication^16,17^. The sex pheromone composition of female *S. frugiperda* includes a blend of compounds, with (*Z*)-9-tetradecenyl acetate (*Z*9-14:OAc) identified as the major sex pheromone component and (*Z*)-7-dodecenyl acetate (*Z*7-12:OAc) as the critical secondary sex pheromone component. The relevance and importance of other compounds in the pheromone gland, such as (*E*)-7-dodecenyl acetate (*E*7-12:OAc), (*Z*)-9-dodecenyl acetate (*Z*9-12:OAc), (*Z*)-10-tetradecenyl acetate (*Z*10-14:OAc), (*Z*)-11-tetradecenyl acetate (*Z*11-14:OAc), and (*Z*)-11-hexadecenyl acetate (*Z*11-16:OAc), remain unclear and may vary depending on the population^14,16,18,19^. For example, while *E*7-12:OAc has been detected in the female sex pheromone blend of *S. frugiperda* populations in Brazil^16^, it has not been reported as an active component in populations from other regions, suggesting possible geographic variation in the sex pheromone composition of this pest. Understanding such geographic variation and its impact on male responses is crucial for the development of efficient and locally adapted pest management strategies, particularly in the context of pheromone-based monitoring, which plays a vital role in sustainable pest management.

The two distinct strains of *S. frugiperda*, known as the 'C' and 'R' strains, may account for the regional variation in female pheromone production and male responses^14^. These strains can be clearly distinguished using mitochondrial *CO1* and nuclear *Tpi* gene markers, while the term 'mitotype' specifically pertains to mitochondrial DNA markers^20–22^. It is known that female C strains found in the USA release a higher amount of *Z*11-16:OAc, whereas female R strains release a higher amount of *Z*7-12:OAc and *Z*9-12:OAc^23^. However, results from Louisiana showed different findings, whereby C-strain females released more *Z*9-14:OAc and less *Z*11-16:OAc than R-strain females^24^. Interestingly, despite variations in pheromone composition, the attraction of males was found to be unaffected by *Z*11-16:OAc^14^. However, C-strain males in some regions responded more strongly to *Z*7-12:OAc than the R-strain males^14^. This indicates that variations in pheromone composition can be influenced by both differences between the strains and the geographic variation within the strains. Furthermore, several other factors, including host plant volatiles, interspecific olfactory cues, and environmental factors such as temperature, humidity, and photoperiod, may contribute to the observed regional variation in sexual communication^25–30^. Such variation in sexual communication reliant on sex pheromones of *S. frugiperda* may impact on their management strategies that depend on effective monitoring of the pest population.

*Spodoptera frugiperda* has recently invaded Africa^31^, spreading rapidly to most sub-Saharan countries^32^, and the presence of both strains has been confirmed on the continent^33–37^. A recent study indicates geographic variation in male moth responses between West African populations (Benin and Nigeria) and populations of *S. frugiperda* in America^38^. However, the extent of this variation remains unclear, and there is no available information regarding East African *S. frugiperda* populations. Furthermore, the introduction of *S. frugiperda* to new regions, including Africa, increased the likelihood of inter-strain mating and gene flow between different populations^39^, which may lead to new combinations of traits. Despite this, the possibility of inter-strain mating between the R and C mitotypes of *S. frugiperda* has not been examined in Africa.

In this study, we hypothesized that there would be variations in the pheromone composition and response of *S. frugiperda* populations in Africa. Additionally, mating would occur between the two mitotypes of *S. frugiperda.* To test these hypotheses, firstly, we identified *S. frugiperda* mitotypes using mtDNA markers from field-collected specimens across populations in Kenya. Secondly, we identified the pheromone compounds present in female glands using GC–MS. Thirdly, we evaluated male moth responses to the pheromone compounds using GC-EAD, EAG, and wind tunnel assays. Finally, we conducted mating and oviposition assays to determine whether R and C mitotype moths would mate and produce eggs. Overall, this study provides valuable insights into sex pheromone communication and mating potential, contributing to the development of effective pheromone-based monitoring and management strategies for *S. frugiperda* in Africa.

## Results

### Genetic identification and distribution of C and R mitotypes of *S. frugiperda*

**Figure 1:**
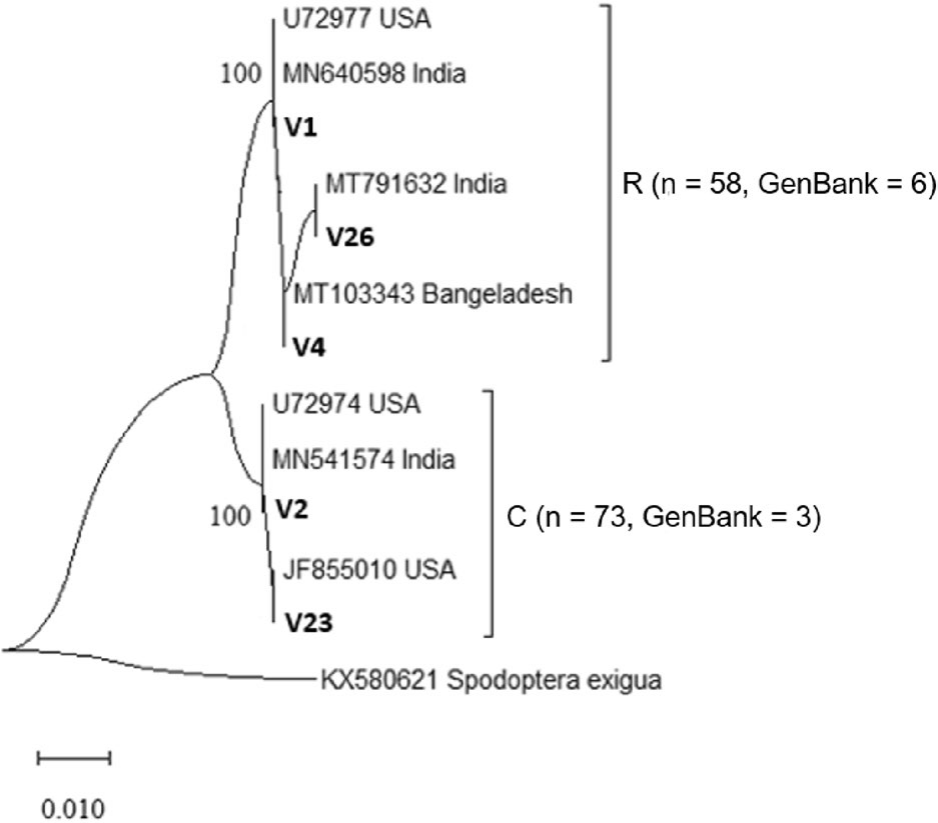
Phylogenetic analysis of *S. frugiperda* based on *mtCOI* sequences from publicly available GenBank and representative haplotypes from this study. The USA R (U72977) and C (U72974) mitotypes are added for reference. V is the representative samples obtained from the study area, while n is the number of individuals classified as belonging to R and C mitotypes. A sequence from *Spodoptera exigua* (KX580621) is included as an out-group. The number of moths belonging to R and C mitotypes, as well as their identity with other samples from publicly available GenBank, are listed in Supplementary Table S1.

**Figure 2:**
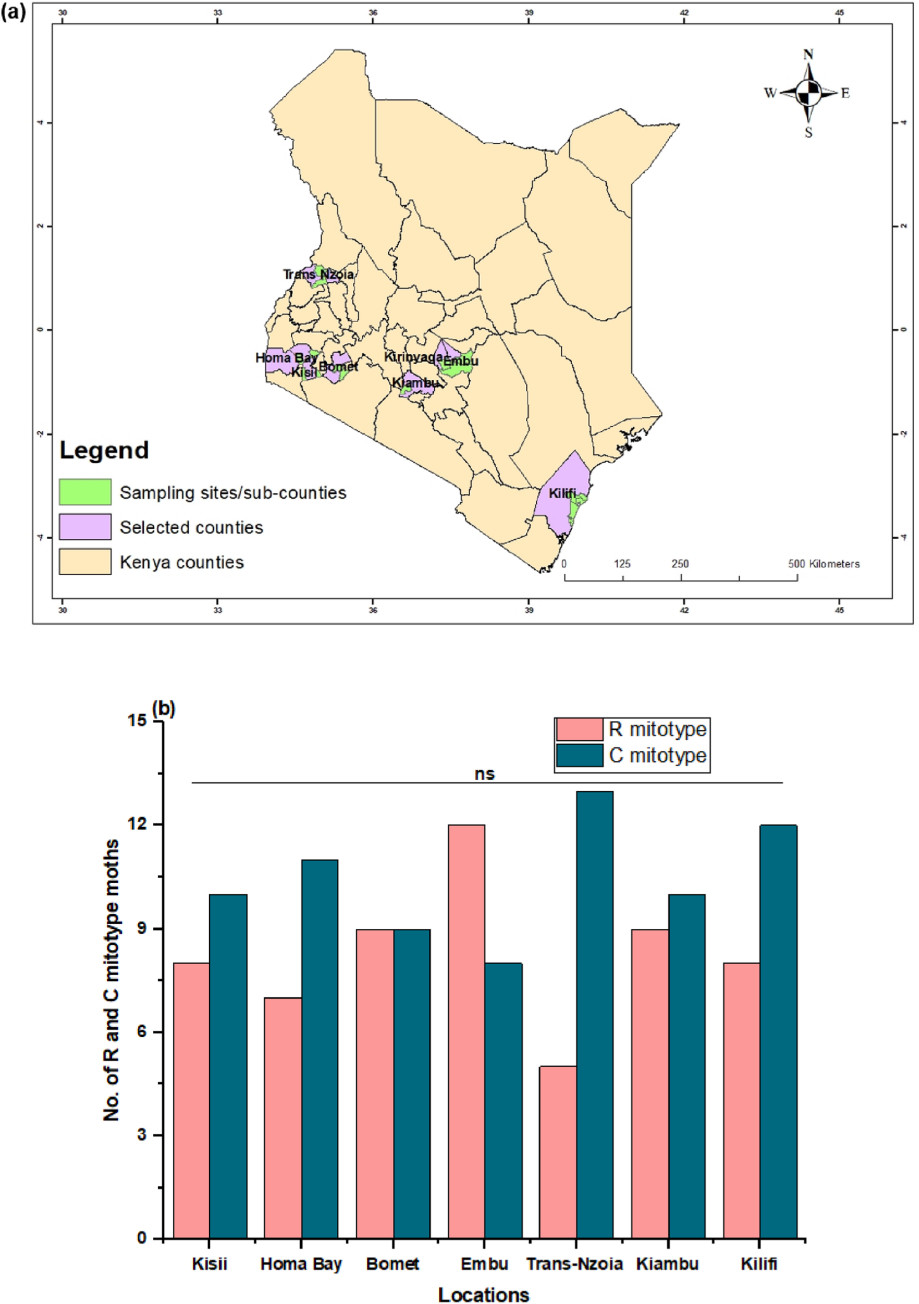
Map showing the *S. frugiperda* collection sites in Kenya (**a**). The figure was generated using QGIS software (version 3.30.2; http://qgis.org). Number of individuals from the R and C (n = 58 and 73, respectively) mitotypes of *S. frugiperda* found in different locations in Kenya (**b**). A Chi-squared test indicated no significant difference (ns) in the frequencies of *S. frugiperda* mitotypes among locations.

Using Lep, LCO 1490, and HCO 2198 markers, a clear separation was found between the C and R mitotypes of sampled *S. frugiperda*. Of all moths collected, 73 belonged to the C mitotype, while the remaining 58 were identified as the R mitotype (Fig. 1). Both the R and C mitotypes of *S. frugiperda* were found in all the sampled locations (Fig. 2a). However, there were no significant differences in the number of R (df = 6, χ^2^ = 3.3, *P* > 0.05) and C (df = 6, χ^2^ = 1.7, *P* > 0.05) mitotypes of *S. frugiperda* found across locations (Fig. 2b).

### Differences in female *S. frugiperda* pheromone production between C and R mitotypes

**Figure 3:**
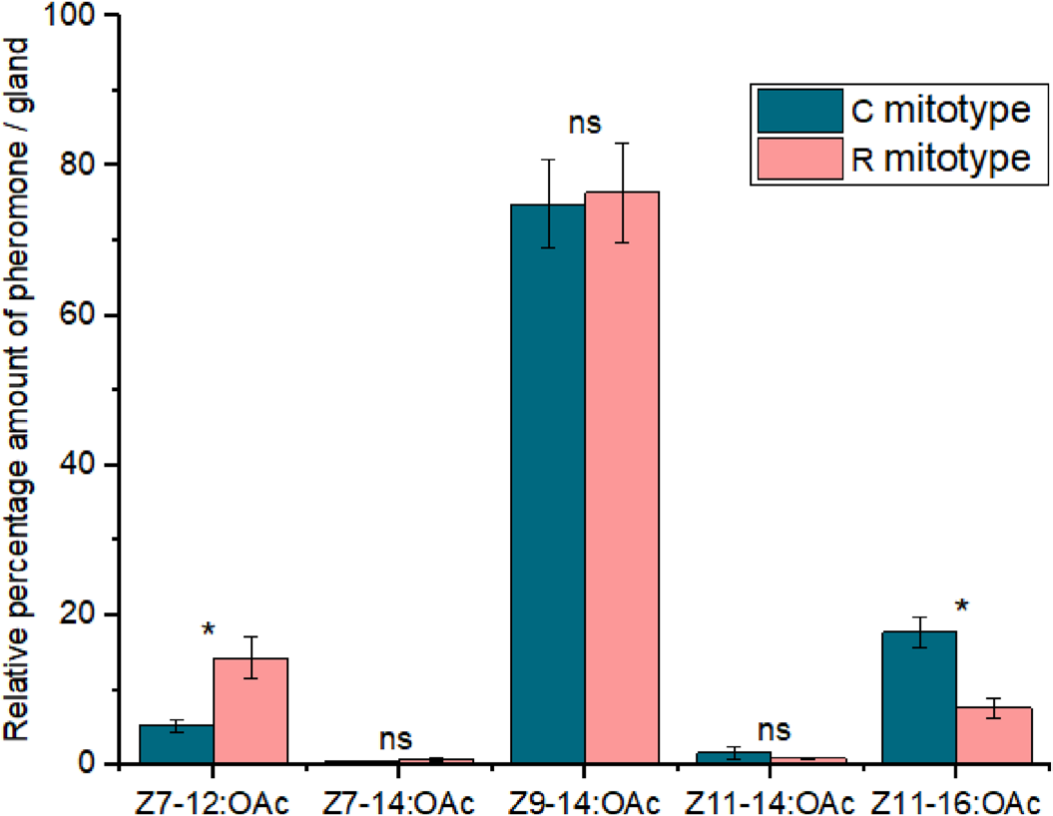
Comparison of relative percentage pheromone composition between the R and C mitotypes of female *S. frugiperda* moths. *ns* not significant, *P < 0.05 using Tukey’s range test. A heatmap showing the relative abundance of compounds in the two mitotypes and their corresponding chromatograms are shown in supplementary Figs. 1 and 2.

Female moths of both the C and R mitotypes were found to have five pheromone compounds in their glands. In both mitotypes, the major sex pheromone component *Z*9-14:OAc exhibited a higher relative percentage than other compounds (Fig. 3). The relative percentage composition of *Z*9-14:OAc was comparable between the mitotypes, and Tukey’s test did not reveal significant differences in its amounts (df = 4, *P* > 0.05). However, the rice mitotype female moths had a 2.7 times higher relative percentage composition of the critical secondary sex pheromone component *Z*7-12:OAc compared to the corn mitotype female moths, while the corn mitotype female moths had a 2.4 times higher relative percentage composition of the compound *Z*11-16:OAc than the rice mitotype female moths. The relative percentage composition of these compounds was significantly different between the two mitotypes (*df* = 4, *P* < 0.05). The two minor compounds, *Z*7-14:OAc and *Z*11-14:OAc, were found in very low relative percentages and Tukey’s test did not detect significant differences in the amounts of these compounds between the two mitotypes (*df* = 4, *P* > 0.05) (Fig. 3).

### GC-EAD analysis

**Figure 4:**
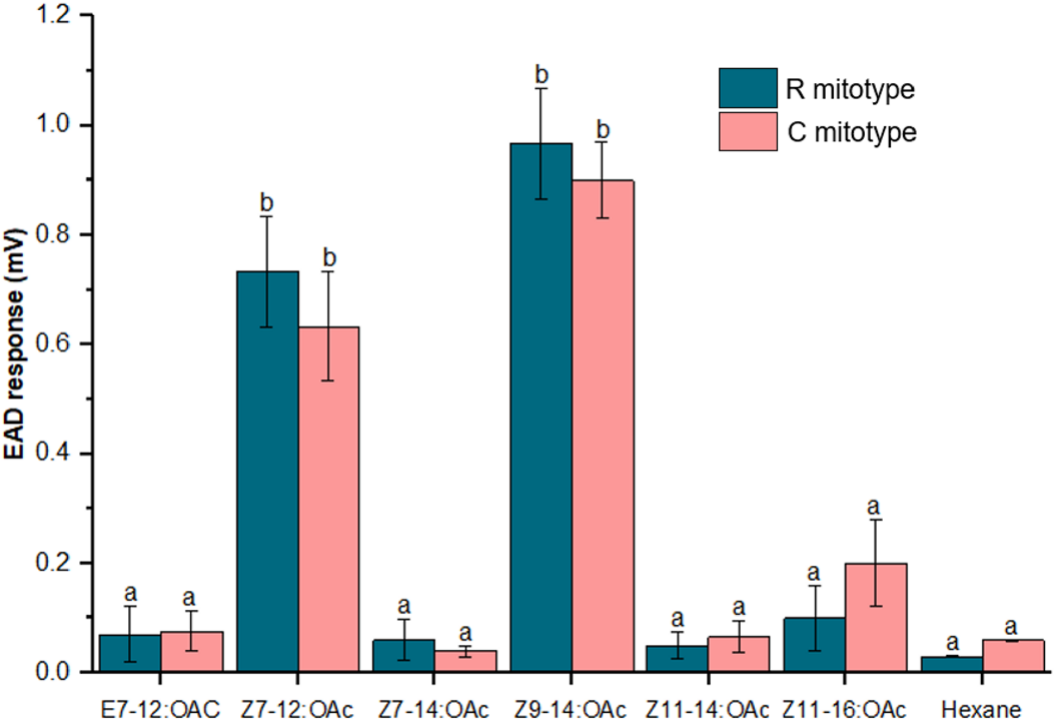
GC-EAD recording of male *S. frugiperda* moths belonging to R (n = 5) and C (n = 5) mitotypes in response to 10 μg of different pheromone compounds (mean ± SE). Bars with different letters are significantly different at *P* ≤ 0.05 using Tukey’s range test.

Both the laboratory-established C and R mitotypes of male *S. frugiperda* exhibited significantly higher EAD responses to *Z*7-12:OAc and *Z*9-14:OAc (*df* = 6, *P* < 0.001) compared to other tested compounds (Fig. 4). There were no significant differences in EAD responses to these two compounds between the two mitotypes (*df* = 6, *P* > 0.05). Moth responses to *E*7-12:OAc, *Z*11-14:OAc, and *Z*11-16:OAc were the lowest in both mitotypes, and there were no significant differences (*df* = 6, *P* > 0.05) in EAD responses to these three compounds between the two mitotypes (Fig. 4).

### EAG responses

**Figure 5:**
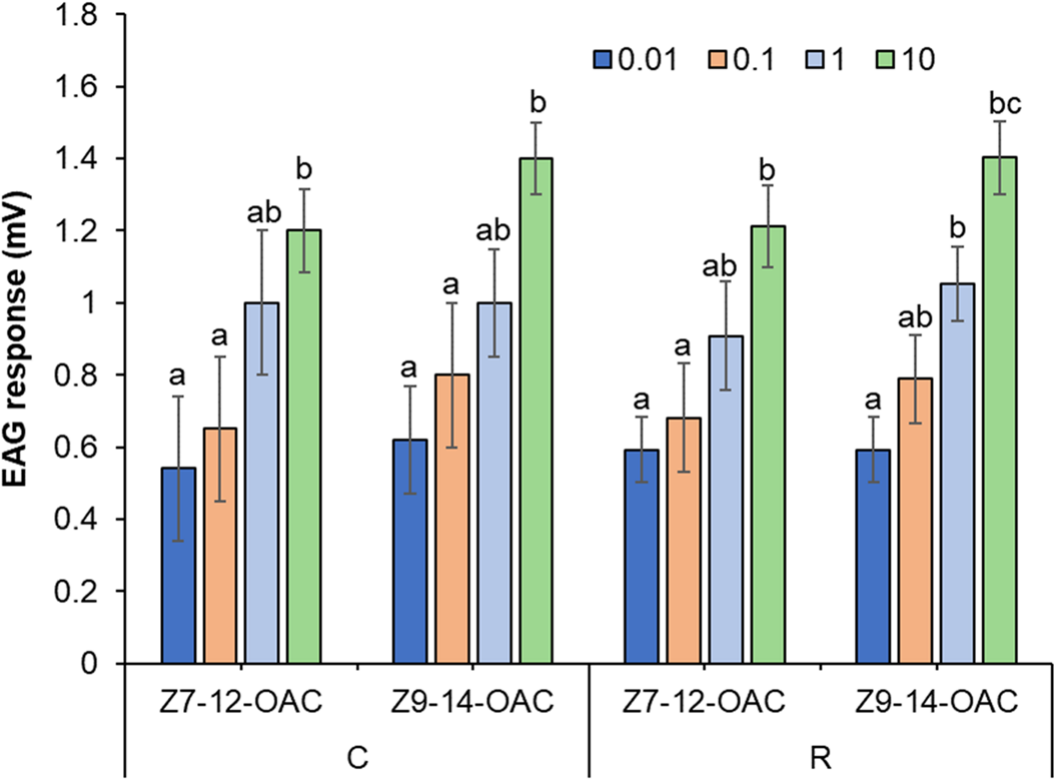
EAG responses of R (n = 10) and C (n = 10) mitotypes of male *S. frugiperda* moth to *Z*7-12:OAc and *Z*9-14:OAc at different doses (mean ± SE). Bars labeled with different letters indicate significant differences at P ≤ 0.05 based on Tukey's range test.

Male *S. frugiperda* moths from both the laboratory-established C and R mitotypes exhibited comparable EAG responses to pheromone compounds (Fig. 5). The moth response increased relatively with the doses of *Z*7-12:OAc and *Z*9-14:OAc. The moth response relatively increased as the doses of *Z*7-12:OAc and *Z*9-14: OAc increased (Fig. 5). However, there were no significant differences in the moth responses to these two compounds between the two mitotypes of male moths, indicating similar responsiveness (*P* > 0.05) (Fig. 5).

### Wind tunnel bioassay

**Figure 6:**
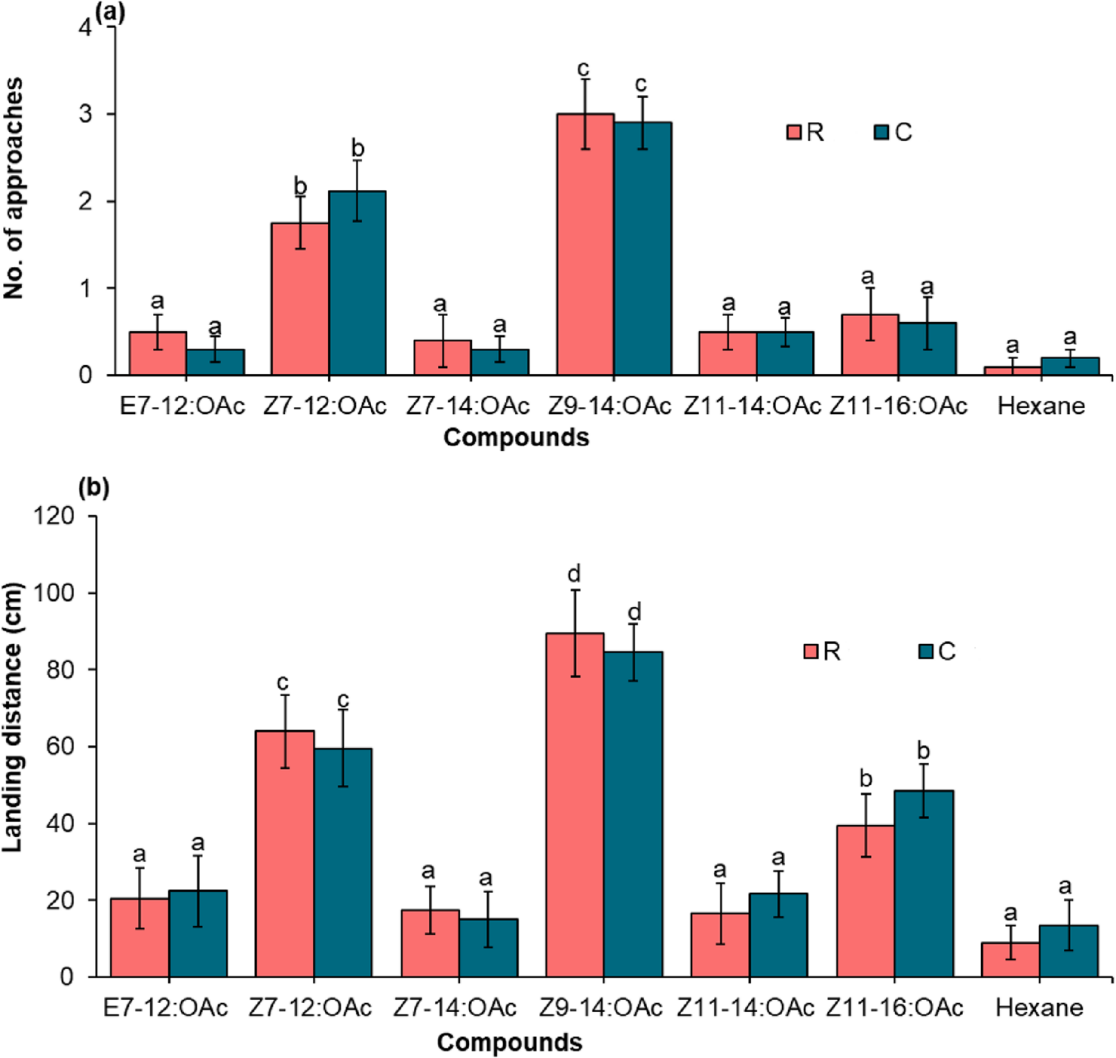
Behavioural responses of R and C mitotypes of *S. frugiperda* moths towards different pheromone compounds in a wind tunnel. The data represent the number of approaches (**a**) and landing responses (**b**) per male tested (n = 10). Bars with different letters are significantly different at p ≤ 0.05 using chi-squared tests.

The responses of male *S. frugiperda* to pheromone compounds varied significantly in a wind tunnel (Fig. 6). Moths of both the laboratory-established C and R mitotypes significantly increased their approaching and landing responses to *Z*9-14:OAc and *Z*7-12:OAc (Fig. 6a, b) (*df* = 6, *P* < 0.001). Lower approaches by both moth mitotypes were observed when they were exposed to *Z*7-14:OAc, *E*7-12:OAc, *Z*11-14:OAc, and *Z*11-16:OAc when compared with the other tested compounds. The landing response of male moths from both mitotypes to *Z*11-16:OAc was significantly higher than to *Z*7-14:OAc, *E*7-12:OAc, and Z11-14:OAc (*df* = 6, *P* < 0.05). On the other hand, male moths of both mitotypes exhibited similarly low landing responses to *Z*7-14:OAc, *E*7-12:OAc, and *Z*11-14:OAc, with no significant differences between them (*df* = 6, *P* > 0.05) (Fig. 6a, b).

### Inter-mitotype mating

**Figure 7:**
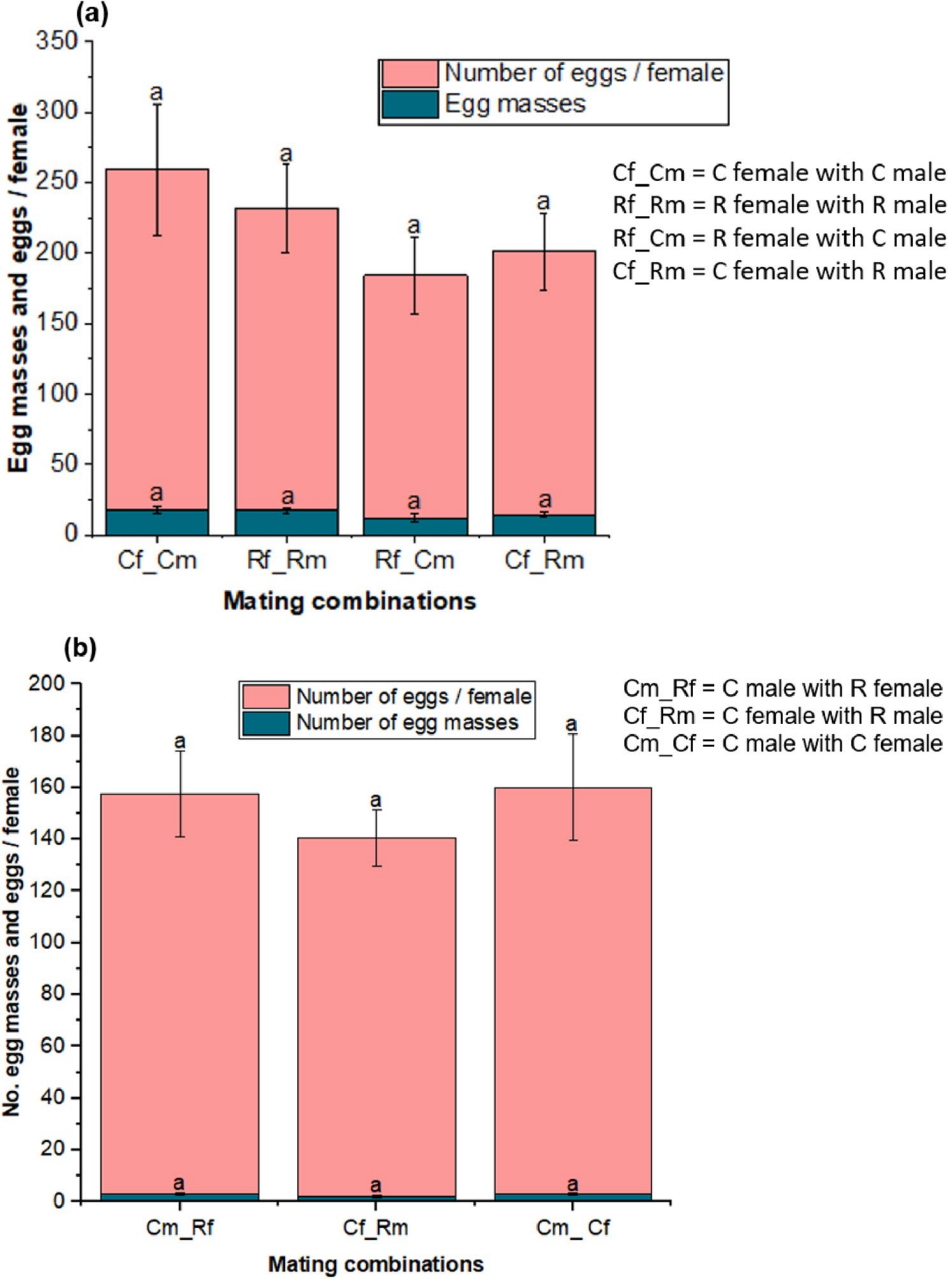
Mean number (± SE) of eggs laid by different combinations of *S. frugiperda* moths from mitotype-specific laboratory colonies (n = 20) (**a**). Mean number (± SE) of eggs laid by different combinations of *S. frugiperda* moths from randomly paired adult moths collected from the field (n = 21) (**b**). Bars with different letters are significantly different at P ≤ 0.05. Phylogenetic analysis of *S. frugiperda* adult pairs that produced eggs is shown in supplementary Fig. 3.

There was egg production in all laboratory reared *S. frugiperda* mitotype combinations, with no statistical differences (*P* > 0.05) between any of the mating combinations (Fig. 7a). DNA analysis of field collections of *S. frugiperda* samples revealed that female moths from the C mitotype mated with male moths from the R mitotype, and likewise, female moths from the R mitotype mated with male moths from the C mitotype (Fig. 7b). The number of eggs produced by these pairs were not significantly different (*P* > 0.05) (Fig. 7b).

## Discussion

In this study, we investigated the potential variation in female moth pheromone composition and male moth response between R and C mitotype *S. frugiperda* populations in Africa, specifically in Kenya. Among the five pheromone compounds identified in the female glands, the major sex pheromone component *Z*9-14:OAc was the most abundant, with a relative percentage composition similar in both mitotypes. On the other hand, the R mitotype females of *S. frugiperda* had a higher relative percentage composition of *Z*7-12:OAc, while the C mitotype female moths had a higher relative percentage composition of *Z*11-16:OAc. This study also confirmed that the *S. frugiperda* populations in Kenya have the same pheromone profiles as the *S. frugiperda* populations in America, which also exhibited higher relative percentage compositions of *Z*7-12:OAc in R strains and *Z*11-16-OAc in C strains^14,19,23^. However, previous studies have reported variation in pheromone composition among different *S. frugiperda* populations. For instance, R strain of *S. frugiperda* from Louisiana R strain had a higher amount of *Z*11-16:OAc, while the C strain had higher amount of *Z*9-14:OAc^24^. Similarly, C strain and R–C hybrid descendant females from Benin and Nigeria produced relatively higher amounts of *Z*9-14:OAc, *Z*7-12:OAc, and *Z*11-16:OAc than *Z*9-12:OAc and *E*7-12:OAc^38^. Other minor compounds were also detected in *S. frugiperda* populations in different regions, such as *Z*9-12:OAc in China, Florida, Brazil, Nigeria, and Benin, *E*7-12:OAc and (*Z*)-10-tetradecenyl acetate in Brazilian *S. frugiperda* populations^16,19,38,40^. Recently, Tabata et al. (2023)^41^ also reported the detection of another key minor compound, (*Z*,*E*)-9,12-tetradecadienyl acetate, in female extracts in Japan. We checked for the presence of *E*7-12:OAc, and results indicated no detectable amounts present in our samples. However, we identified a minor compound, *Z*7-14:OAc, in the female glands, which had not previously been reported in *S. frugiperda* moths but had been detected in several other lepidopteran insects^42–44^. Further research is required to determine whether this compound is a pheromone component because some compounds can be detected in the female gland but may not constitute the pheromone blend emitted by females, and hence male moths may not respond to it.

Our results from the GC-EAD and EAG experiments provide important insights into the pheromonal compounds responsible for male attraction in S*. frugiperda* moths. The fact that both C and R mitotypes of male moths had higher antennal responses to *Z*9-14:OAc and *Z*7-12:OAc suggests that these two compounds are important bioactive components of the female pheromone blend. Additionally, the dose–response study using EAG revealed that male moth responses increased as the doses of *Z*9-14:OAc and *Z*7-12:OAc increased. This underscores the significance of identifying key pheromone compounds and determining the detectable amounts by target insects to develop effective pest management strategies. Similar to our findings, higher antennal responses to *Z*9-14:OAc were observed in *S. frugiperda* moths from Florida^38^, and weak antennal responses to *Z*11-16OAc were observed in *S. frugiperda* populations from China, Florida and Brazil^38,45^. However, it is important to note that there are variation in the response of different *S. frugiperda* populations to these compounds. For example, *E*7-12:OAc evoked weak antennal responses in Kenya *S. frugiperda* populations in our study, although it elicited higher antennal responses in the *S. frugiperda* populations from Mexico and Brazil^16,38^. Additionally, C-strain males in Florida and Puerto Rico showed a higher response to *Z*7-12:OAc than R-strain males^14^.

Our wind tunnel bioassay produced results that were consistent with those obtained using GC-EAD and EAG techniques. We observed that males from both moth mitotypes exhibited a greater level of attraction towards *Z*9-14:OAc and *Z*7-12:OAc compared to the other compounds tested in the wind tunnel. Both moth mitotypes showed a higher frequency of approach and landing responses towards *Z*9-14:OAc and *Z*7-12:OAc relative to the other compounds tested. According to Wang et al. (2022)^45^, *Z*9-14:OAc alone induces male sexual behaviours in a wind tunnel, including flying, upwind anemotaxis, approach, and landing. Similarly, *Z*9-14:OAc showed strong attraction to both strains of *S. frugiperda* in Puerto Rico^14^. In Costa Rica, *Z*7-12:OAc and *Z*9-12:OAc were highly attractive to *S. frugiperda* when presented alone^46^. On the other hand, Cruz Esteban et al. (2020)^47^ observed higher moth attraction to blends of *Z*7-12:OAc + *Z*9-14:OAc than to single compounds in Mexico. We found the attraction of male moths of both mitotypes to *Z*11-16:OAc was low and not clear in the wind tunnel, which has also been observed by Cruz Esteban et al. (2020)^47^ in Mexico. Moreover, *E*7-12:OAc, *Z*7-14:OAc, and *Z*11-14:OAc did not attract male moths of both mitotypes, and *E*7-12:OAc was not an essential component for S*. frugiperda* sexual communication in China^40^. However, these compounds may not attract males by themselves but can significantly enhance attraction when added to the two critical sex pheromone components, *Z*7-12:OAc and *Z*9-14:OAc. It is crucial to consider the potential synergistic effects constituent components rather than testing single compounds. Furthermore, olfactory responses of *S. frugiperda* to pheromone blends may be influenced by various factors, including background host plant volatiles, interspecific olfactory cues, and environmental factors such as temperature, humidity, and photoperiod, that may vary geographically. Future work should consider examining the effect of these factors on the pheromone composition and behavioral responses of *S. frugiperda*.

Our study showed that mating between the two identified mitotypes of *S. frugiperda* results in comparable egg production to that observed within the same mitotype. Even though the sample size was small, this results imply that hybrid offsprings with intermediate characteristics between the two mitotypes or expressing the dominant gene of one of the mitotypes can be produced. Our findings are consistent with recent studies that have reported evidence of hybridization between C and R strain populations in Africa, which has led to the existence of genetically hybrid *S. frugiperda* populations^48–51^. These results have important implications for the genetic diversity and potential spread of *S. frugiperda* populations in Africa. It highlights the need for further investigation into the effects of hybridization on the biology and ecology of this pest species. However, we recommend further research using Tpi genes to enhance the accuracy of strain identification, as mitochondrial markers alone may not reliably distinguish the two strains.

## Conclusion

In conclusion, our study provides important insights into the pheromone communication system of *S. frugiperda* in Africa, and our findings have practical implications for developing effective pest monitoring and management strategies. The high attraction of both mitotypes to *Z*9-14:OAc and *Z*7-12:OAc highlights the potential of these compounds to develop pheromone-based management techniques. Moreover, our study demonstrated that the *S. frugiperda* population in Kenya has the same pheromone profile as the American *S. frugiperda*, which suggests that pheromone-based management strategies developed for *S. frugiperda* in America could be applicable to African populations. However, mating between C and R mitotypes of *S. frugiperda* populations in Africa raises questions about the potential impact of hybridization on pest management strategies. Therefore, further research is needed to fully understand the complex interactions between different pheromone compounds and male moth response, as well as the potential differences in pheromone production and male response between hybrid and pure mitotypes (R and C mitotypes). Overall, our findings not only provide a basis for further research but also offer valuable insights for the development of more effective and sustainable pheromone-based management strategies for *S. frugiperda* populations in Africa.

## Materials and methods

### *Spodoptera frugiperda* sampling and colony establishment

Third and fourth instar larvae were collected from maize plants in various locations within *S. frugiperda* affected areas in Kenya (Fig. 2a) and reared under controlled laboratory conditions (24 ± 4 °C, 60 ± 5% RH, and a 12L:12D photoperiod). The larvae were placed in vials (30 ml) and fed an artificial diet as described in Prasanna et al. (2018)^52^. The sex of the pupae was identified by observing the identifying features of the terminal segments using a 10 × Leica EZ4 HD stereo microscope (Leica Microsystems, Wetzlar, Germany). Female pupae have a wider gap between the genital and anal openings than male pupae^53^. A single pair of identified sexed pupae was put inside a Petri dish in an oviposition cage (20 cm × 20 cm 20 cm). Newly emerged adults were fed using a cotton ball soaked in a 10% honey-water solution, and wax paper was placed inside the oviposition cage as an egg laying substrate. Adult pairs that produced fertile offspring were genetically analysed to identify their mitotype, and the offspring were labelled and reared on artificial diet to establish a mitotype-specific laboratory colony. Only the offspring resulting from the mating of male and female parents belonging to the same mitotype were used to establish a mitotype-specific laboratory colony. The second to fourth laboratory generations were used for the pheromone extraction, electrophysiological and wind tunnel assays.

### Mitotypes identification

The Isolate II Genomic DNA Kit (Bioline, London, United Kingdom) was used to extract genomic DNA from individual insects, following the method described by Gichuhi et al. (2020)^33^. The DNA was eluted in a final volume of 50 μl and quality and quantity checks were performed with the Nanodrop 2000/2000c Spectrophotometer (Thermo Fisher Scientific, Wilmington, USA). Polymerase chain reaction (PCR) was done to amplify the mitochondrial COI gene using LepF1 5′ ATTCAACCAATCATAAAGATATTGG 3′ and LepR1 5′ TAAACTTCTGGATGTCCAAAAAATCA 3′ ^54^ markers in addition to the traditional barcode region markers LCO 1490 5′ GGTCAACAAATCATAAAGATATTGG 3′ and HCO 2198 5′ TAAACTTCAGGGTGACCAAAAAATCA 3′ ^55^. The PCRs were performed in a 20 µL volume containing 5X My *Taq* Reaction Buffer (5 mM dNTPs, 15 mM MgCl_2_, stabilizers and enhancers), 0.5 pmol µl^-1^ of each primer, 0.5 mM MgCl_2_, 0.0625 U µl^−1^ My *Taq* DNA polymerase (Bioline, London, UK) and 15 ng µl^−1^ of DNA template. The reactions were set up in the Nexus Mastercycler gradient (Eppendorf, Germany) with the following cycling conditions: initial denaturation for 2 min at 95 °C, followed by 40 cycles of 30 s at 95 °C, 30 s annealing (52 °C for LepF1/R1 and 54.1 °C for LCO/HCO), extension for 1 min at 72 °C, then a final elongation step of 10 min at 72 °C. The PCR-amplified products were separated on a 1.2% agarose gel. KETA GL imaging system trans-illuminator was utilized to analyse and record DNA bands on the gel (Wealtec Corp, Meadowvale Way Sparks, Nevada, USA). Following the manufacturer's instructions, successively amplified products were excised and purified using the Isolate II PCR and Gel Kit (Bioline, London, UK). Purified samples were sent to Macrogen Europe BV (Meibergreef, Amsterdam, the Netherlands) for bi-directional sequencing.

### Sequence analyses

Geneious Version 8 (http://www.geneious.com) was used to assemble and edit the successful sequences^56^. The primer sequences were removed from the consensus sequences derived from both the forward and reverse reads, resulting in consensus sequences with a length of approximately 608 base pairs. Similarity searches were performed by querying the consensus sequences via Basic Local Alignment Search Tool (BLAST) at the GenBank database hosted by the National Centre for Biotechnology Information (NCBI) to confirm the species' identity. The BLAST algorithm searches for regions of local similarity between sequences by comparing consensus sequences to reference sequences in the GenBank database. In addition, the query was conducted in BOLD (Barcode of Life Database). MEGA version X was used for phylogenetic and molecular evolutionary analyses^57^ using the Maximum Likelihood method based on the Tamura-Nei model^58^. The tree's reliability was assessed using 1000 bootstrap replications.

### Pheromone extraction and analysis

Virgin female moths (2 to 3 days old) exhibiting various behaviours to call their mates, such as wing fanning and extruding the tip of the abdomen. Female exhibiting such behaviours were selected for pheromone extraction. During the first two to four hours of the dark period, the abdominal tip (between the 8th and 9th abdominal segments) of moths was gently excised. The excised glands were placed individually into a glass vial with 50 μl hexane. After 30 min, the gland was removed from the solvent, and the extracts were kept at −20 °C until used for GC–MS analysis. The pheromone extract samples were analyzed using an Agilent 5975/6880 GC–MS, with both DB-Wax (60 m × 0.25 mm × 0.25 μm; Agilent) and an HP-5 MS (30 m × 0.25 mm × 0.25 μm, Agilent) columns. Helium was used as the carrier gas at 1.2 ml/min. The oven temperature was kept at 35 °C for 5 min and then increased to 280 °C at a rate of 10 °C per minute. The pheromone samples (2 μl) from the original 50 μl hexane extracts were directly injected into the GC using an autosampler. Retention times were converted to retention indices relative to the retention times of n-alkanes standards according to Adams (1996)^59^ and Babushok (2015)^60^. The compounds were tentatively identified by comparing mass spectra and the Kovats retention index to the Adams library and NIST databases. For confirmation, a comparison of the retention times, retention indices and mass spectra of authentic standards run under the same conditions was carried out. All compounds found in the control were regarded as contaminants and thus ignored during identification.

### GC-EAD analysis

This experiment examined the moth antennal responses to pheromone compounds to identify the compounds that elicited the greatest antennal response. The male moth responses to the five pheromone compounds identified from the female gland of *S. frugiperda* (*Z*7-12:OAc, *Z*7-14:OAc, *Z*9-14:OAc, *Z*11-14:OAc, and *Z*11-16:OAc) were studied. *E*7-12:OAc, which has been discovered in Brazilian *S. frugiperda* populations, was included to determine whether it elicited antennal responses in *S. frugiperda* populations from Africa. The synthetic standard of these sex pheromone compounds (90% purity) were purchased from Alfa Chemistry (New York, USA). Electroantennogram detection responses to pheromones were recorded using GC-EAD 2011 (V.1.2.3, Syntech, Kirchzarten, Germany) and an Agilent Technologies 6890 GC (Santa Clara, CA, USA) equipped with a DB-Wax capillary column (60 m × 0.25 mm × 0.25 μm; Agilent). The GC oven temperature programmed from 40 °C for 3 min, 10°Cmin^−1^ to 240 °C (held for 5 min). Helium was the carrier gas at 1.2 ml min^−1^. The antennae of 3–5-day-old unmated males were clipped at the base of flagella segments with micro scissors, and the tips were also clipped. The basal part of the antenna was inserted into a glass electrode filled with Beadle-Ephrussi Ringer solution as the reference electrode. The last one to two distal ends of antennal segments were inserted into the tip of the recording glass capillary electrode. The signals generated by the antenna were passed through a high-impedance amplifier (NL1200; Syntech, Hilversum, Netherlands). An antenna was stimulated with 10 μg of each synthetic pheromone compound that was dissolved in 2 μl of hexane and injected into the GC. Five moth antennae from each mitotype were tested for each compound.

### Pheromone dose response study

EAG recordings of synthetic blends containing *Z*7-12:OAc and *Z*9-14:OAc (compounds that elicited the greatest antennal response in GC-EAD analysis) were evaluated at doses of 0.01, 0.1, 1, and 10 μg, using the antennae of 3 to 5-day-old unmated male *S. frugiperda*. Adult moths were monitored as described above. Serial dilutions of the synthetic compounds were prepared in hexane to create 0.01, 0.1, 1, and 10 μg solutions.

### Wind tunnel bioassay

The attraction of male *S. frugiperda* moths to female sex pheromone was studied using an aluminium framed wind tunnel (120 × 32 × 32 cm). The experiments were conducted at 25 ± 2 °C and 60 ± 10% relative humidity, while air speed inside the wind tunnel was 20 cm/s. The responses of male moths to single doses (10 μg) of pheromone compounds were examined. Each compound dissolved in 10 μl of hexane was pipetted onto a small piece of filter paper (1 cm^2^) and placed outside the wind tunnel in a glass cylinder with quick-fit connection on both ends using a portable volatile collection kit (B.J. Pye, Hertfordshire, UK). Volatiles were drawn from the container using charcoal-filtered air at a rate of 500 ml min^−1^ a vacuum pump. Moths were acclimatized to the wind tunnel room conditions 1 h before testing. Unmated male moths (3–5 days old) were carefully introduced individually through a hole located at the top of the wind tunnel, 120 cm away from the odour source during 2 to 4 h within the scotophase. A total of ten moths of each mitotype were tested for individual compounds. A light source provided by a bulb (40 Watt, fitted with red filter) positioned at 50 cm above the tunnel was used to observe moth movement. The moth responses, such as the number of approaches (20 cm from the pheromone source) and flight distance (the distance from the release point to where the moth ultimately settled after approaching the odour source), were recorded visually. Moths that did not respond within 5 min were discarded. Filter paper impregnated with 10 μl hexane was included as controls for all experiments.

### Mating between C and R mitotypes

Two experiments were conducted to determine whether R and C mitotype moths’ could mate and produce eggs. In the first experiment, an oviposition test was conducted using a known *S. frugiperda* (mitotype-specific) colony. Pupae were sexed and monitored as described above. A single pair of sexed adult moths from mitotype-specfic colonies was allowed to lay eggs on wax paper in an oviposition cage. The moths were combined as described below (Table 1). Five pairs of *S. frugiperda* moths were tested from each combination. Leica EZ4 HD stereo microscope (Leica Microsystems, Wetzlar, Germany) with a 16× magnification was used to count the egg masses and number of eggs per mass.

**Table 1:** Mating combinations of *S. frugiperda* mitotypes.

Mating pairs	Sex	Mitotypes
1	Female	C
	Male	C
2	Female	R
	Male	R
3	Female	C
	Male	R
4	Female	R
	Male	C

In the second experiment, unknown mitotypes of *S. frugiperda* moths were used due to the possibility of greater genetic diversity in moths collected from the field as compared to the controlled, known mitotypes used in the first experiment. The larvae were collected from different locations and monitored as described above. Pupae from field collections and unknown mitotypes were randomly selected and subsequently sexed using the method described above. A single pair of sexed moths was allowed to lay eggs on wax paper placed inside the oviposition cage. After oviposition, all adult pairs that produced eggs were genetically analysed to identify their mitotype. Eggs and egg mass produced from randomly sexed pupae and molecularly identified mating pairs of C male (Cm) vs. R female (Rf), C female (Cf) vs. R male (Rm), and C male (Cm) vs. C female (Cf) were counted to determine whether mating pairs of R–C mitotypes differed in their ability to produce eggs. By chance, during the random selection and sexing of pupae from field collections, no pupae of R mitotype males (Rm) and R mitotype females (Rf) were selected. Consequently, no eggs produced from this specific combination. A total of seven pairs of moths were used for this experiment.

### Data analysis

The normality and homogeneity of all data were checked using the Shapiro–Wilk and Bartlett tests, respectively. The Chi-squared test was used to analyze the number of R and C mitotypes of *S. frugiperda* found across different locations, as well as the moth approaches and landing responses in the wind tunnel. All the pheromone compounds found in 50 female moths of each mitotype were converted into relative percentages, and a heatmap clustering analysis (supplementary material) was used to identify the most abundant compounds in the female gland. Further analysis was conducted to examine whether there were differences in the relative percentages of pheromone composition between the R and C mitotypes using analysis of variance (ANOVA). The spike amplitude (mV) of GC-EAD and EAG responses were transformed using log (x + 1) to meet assumptions of normality and homogeneity and subjected to a one-way analysis of variance. Tukey's range test (p ≤ 0.05) was used to compare the mean values of the data. Due to data overdispersion, egg counts were analysed using a generalized linear model (GLM) with a negative binomial distribution. The number of *S. frugiperda* eggs laid by each mitotype combination was used as the response variable, and mean values of the data were compared using the 'emmeans' package. R statistical software version 4.0.4 was used for all statistical analyses.

### Supplementary Information

Supplementary Figures.

Supplementary Table S1.

## Data Availability

The raw data generated during the current study are available from the corresponding author upon reasonable request.
