# Supplementary material for: Pheromonal variation and mating between two mitotypes of fall armyworm (Spodoptera frugiperda) in Africa
Source: Sci Rep. 2024 Feb 15;14:3848. doi: 10.1038/s41598-024-53053-9 (PMC10869808; doi:10.1038/s41598-024-53053-9)

**Supplementary Figure 1.** Heatmap indicating the relative abundance of compounds in the sex pheromone of female *S. frugiperda* moths of the R (n = 50) and C (n = 50) mitotypes


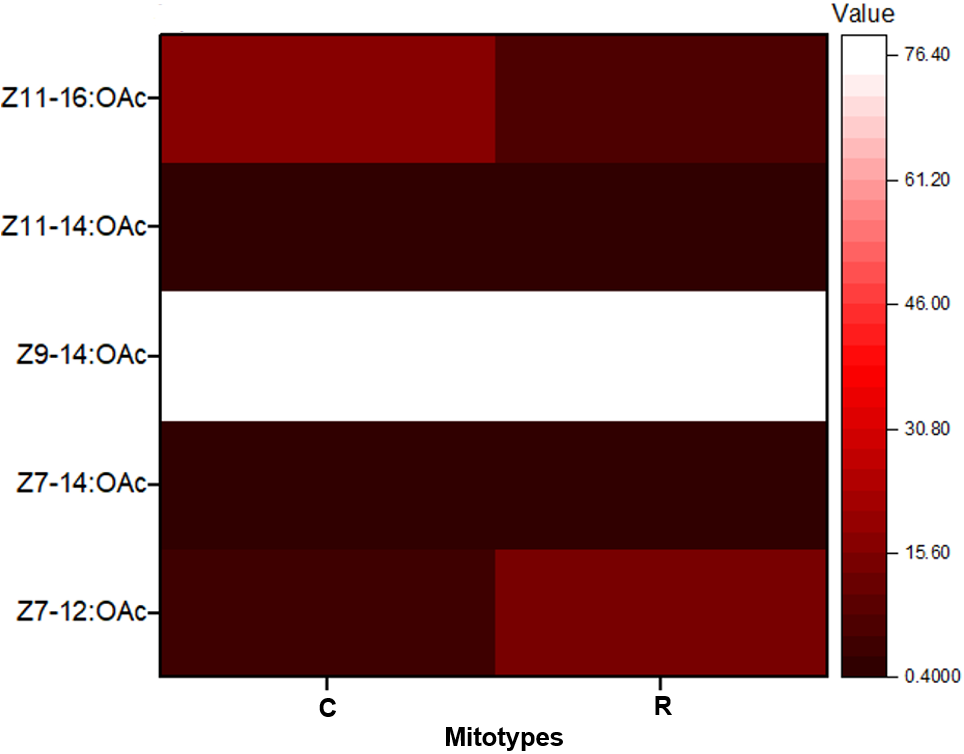


**Supplementary Figure 2:** Gas chromatography mass spectrometry (GC-MS) chromatograms of pheromone compounds. Peak 1 represents Z7-12:OAc, Peak 2 corresponds to Z7-14:OAc, Peak 3 to Z9-14:OAc, Peak 4 to Z11-14:OAc, and Peak 5 to Z11-16:OAc


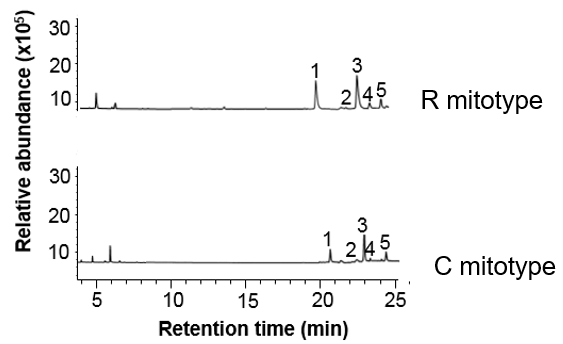


**Supplementary Figure 3.** Phylogenetic analysis of *S. frugiperda* adult pairs that produced eggs, based on mtCOI sequences obtained from the GenBank (<https://blast.ncbi.nlm.nih.gov/Blast.cgi>). An out-group sequence from *Spodoptera exigua* (KX580621) was included. The USA C (U72974) and R (U72977) strains were used as reference sequences (a). The female and male moths are indicated as FM and MM, respectively, and each pair of moths is represented by similar numbers.


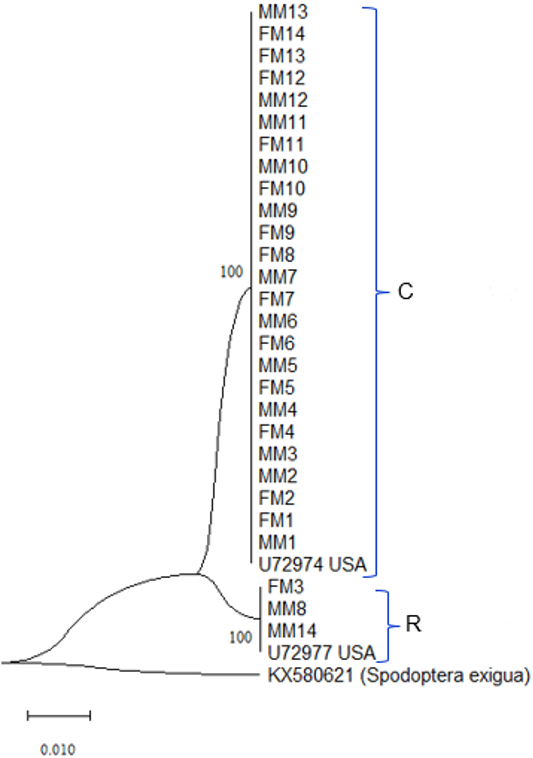

Supplement: Supplementary file 1 — Supplementary Figures. [file 41598_2024_53053_MOESM1_ESM.docx]
