# Supplementary material for: Pheromonal variation and mating between two mitotypes of fall armyworm (Spodoptera frugiperda) in Africa
Source: Sci Rep. 2024 Feb 15;14:3848. doi: 10.1038/s41598-024-53053-9 (PMC10869808; doi:10.1038/s41598-024-53053-9)
Supplement: Supplementary file 2 — Supplementary Table S1. [file 41598_2024_53053_MOESM2_ESM.pdf]

Supplementary Table S1. The number of moths belonging to R (n = 58) and C (n = 73) mitotypes, as well as their identity with other samples from publicly available GenBank, are listed.

**Mitotypes**	**Samples from GenBank**	**Samples from this study**
**R**	MN640598	V3, V10, V19, V21, V27, V32, V63-V69, V75, V76, V81, V91-V94, V99, V100, V105, V107, V109-V111, V114, V115, V117, V119, R73
	MT103343	V4, V18, V20, V48, V78, V83, V85-V88, V95, V96, V113
	OP132904	V1, V11, V33, V43, V51, V82, V97
	MT180097	V45, V80, V116
	MT791632	V26, V84
	ON797293	V60
**C**	MN541574	V2, V5-9, V12-15, V22, V24, V25, V28-31, V35, V37, V39, V41, V42, V44, V46, V47, V49, V50, V52-V59, V61, V62, V70-V74, V77, V89, V90, V98, V101-103, V106, V118, V120, R52, R53, R55-V57, R59, R63, R65, R69-72, R74-V78, R81, R83
	JF855010	V23
	MT152731	V36
